# A Tribute

**Published:** 1914-06

**Authors:** Victoria A. H. Duggan


### A Tribute.

BY VICTORIA A. H. DUGGAN.

"Doctor Daniel is dead," so the last message read;
"Dead," yet it seems like a dream,
A dream of the Past, which will always last,
Of a man, of the "old régime."

Of the old régime, when woman was queen,
And conquered all doubt and all fear,
By the light of her eye, a tear, or a sigh,
And men, were the conquered, my dear.

"Dead?" no, he lives, as does one who gives,
Of himself, of his heart, and his brain,
His "friends," were as "brothers," for to "do unto others,"
Was the precept he strove to sustain.

His life was replete, with services sweet,
That he counted it joy to extend,
Whether country or friend raised the cry, "come, defend,"
He was there to sustain to the end!

Then lay him away, in his "loved coat of gray,
With his sword and his saddle bags worn,
For he's tired, must rest, from answering quests,
Yet will meet us again, in the Dawn.

